# *p*‐Synephrine, ephedrine, *p*‐octopamine and *m*‐synephrine: Comparative mechanistic, physiological and pharmacological properties

**DOI:** 10.1002/ptr.6649

**Published:** 2020-02-26

**Authors:** Sidney J. Stohs, Mohd Shara, Sidhartha D. Ray

**Affiliations:** ^1^ School of Pharmacy and Health Professions, Creighton University Medical Center Omaha Nebraska; ^2^ Department of Pharmaceutical & Biomedical Sciences, Kitsto Consulting LLC Frisco Texas; ^3^ Faculty of Pharmacy Jordan University of Science and Technology Irbid Jordan; ^4^ College of Pharmacy Touro University Manhattan New York

**Keywords:** adrenergic receptors, ephedrine, *m*‐synephrine, *p*‐octopamine, *p*‐synephrine, trace amine‐associated receptors

## Abstract

Confusion and misunderstanding exist regarding the lack of cardiovascular and other adverse health effects of *p*‐synephrine and *p*‐octopamine relative to ephedrine and *m*‐synephrine (phenylephrine) which are known for their effects on the cardiovascular system. These four molecules have some structural similarities. However, the structural and stereochemical differences of *p*‐synephrine and *p‐*octopamine as related to ephedrine and *m*‐synephrine result in markedly different adrenergic receptor binding characteristics as well as other mechanistic differences which are reviewed. *p*‐Synephrine and *p‐*octopamine exhibit little binding to α‐1, α‐2, β‐1 and β‐2 adrenergic receptors, nor are they known to exhibit indirect actions leading to an increase in available levels of endogenous norepinephrine and epinephrine at commonly used doses. The relative absence of these mechanistic actions provides an explanation for their lack of production of cardiovascular effects at commonly used oral doses as compared to ephedrine and *m*‐synephrine. As a consequence, the effects of ephedrine and *m*‐synephrine cannot be directly extrapolated to *p*‐synephrine and *p*‐octopamine which exhibit significantly different pharmacokinetic, and physiological/pharmacological properties. These conclusions are supported by human, animal and in vitro studies that are discussed.

## 1 INTRODUCTION

The cardiovascular effects of ephedrine and *m*‐synephrine (phenylephrine) are well known. Both ephedrine and *m*‐synephrine have been used to treat various causes of hypotension (Dusitkasem, Herndon, Stahl, Bitticker, & Coffman, 2017). Increases in systolic blood pressure and decreases in heart rate have been reported with oral doses of *m*‐synephrine over 15 mg, and approximately a 20 mmHg increase in systolic blood pressure may occur with an oral dose of 45 mg (Atkinson, Potts, & Anderson, 2015). Typical intravenous doses in the range of 0.7–1.0 mg/kg of ephedrine have been used to treat hypotension (Dusitkasem et al., 2017). Due to adverse effects of ephedrine as tachycardia and palpitations at oral doses as low as 20 mg (Hackman et al., 2006; Haller & Benowitz, 2000), the use of ephedrine‐containing dietary supplements was prohibited by the U.S. FDA in 2004.

Questions have been raised regarding the safety of *p*‐synephrine due to its structural similarities to ephedrine. Various authors have assumed that *p‐*synephrine exhibits the same cardiovascular effects as ephedrine. This conclusion is not supported by approximately 30 peer reviewed human clinical studies that have shown p‐synephrine to be without cardiovascular or other adverse effects at commonly used doses in the range of 25–100 mg per day (Stohs, 2017; Suntar, Khan, Patel, Celano, & Rastrelli, 2018).

It has been widely assumed but without evidence that *p*‐synephrine may act as a stimulant when consumed orally and thus may exhibit cardiovascular activity (Anon, 2010; Bakhyia, Dusemund, et al., 2017, Bakhyia, Ziegenhagen, et al., 2017; Bent, Padula, & Neuhaus, 2004; Fugh‐Berman & Myers, 2004; Haaz, Williams, Fontaine, & Allison, 2010; Health Performance Resource Center, 2015; Inchiosa Jr., 2011; National Center for Complementary and Integrative Health, 2015; Natural Medicines Comprehensive Database, 2016; OPPS, 2016; Penzak et al., 2001; Rasmussen & Keizers, 2015). However, none of these reports critically reviewed the mechanistic studies or the human clinical studies that have been conducted. The current review addresses extant information regarding the comparative mechanisms and effects of ephedrine, *p‐*synephrine, *m*‐synephrine and *p‐*octopamine.

**Figure 1:**
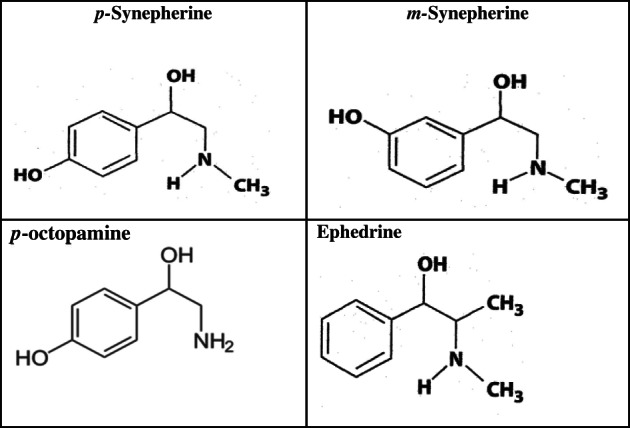
Chemical structures of *p‐*synephrine, *m*‐synephrine, *p*‐octopamine and ephedrine

Chemically and structurally, *p‐*synephrine, *p‐*octopamine (nor‐synephrine) and *m*‐synephrine (phenylephrine) are similar to ephedrine (Figure 1). Ephedrine is a phenylpropanolamine derivative and does not contain a para‐substituted hydroxy group. *p*‐Synephrine and *p*‐octopamine are phenylethanolamine derivatives with a para‐substituted hydroxyl group. *p‐*Octopamine is the *N*‐demethylated derivative of *p*‐synephrine. As will be discussed, these two chemical differences greatly change the stereochemistry, and alter adrenergic receptor binding characteristics and pharmacokinetic properties.

Confusion exists in the literature because there is also *m*‐synephrine (phenylephrine) that possesses the hydroxyl group in the meta‐position on the benzene ring as opposed to the para‐position for *p*‐synephrine and *p*‐octopamine (Figure 1). *m*‐Synephrine is a Food and Drug Administration (FDA)‐approved over‐the‐counter synthetic drug ingredient used in nasal sprays and decongestants.

## 2 SOURCES AND EXPOSURE

The most common commercial source of *p*‐synephrine is *Citrus aurantium* L (bitter orange). The commercial growing of bitter orange began around Seville, Spain in the 12th century and bitter orange was the only citrus grown in Europe for 500 years. Bitter oranges are cultivated extensively throughout the Mediterranean, China, India, Africa, the Middle East, the West Indies and Brazil Humans are widely exposed to varied concentrations of *p*‐synephrine on a daily basis from various juices, and food and beverage (orange flavored liqueurs) products from bitter orange, as well as Marrs sweet oranges (*Citrus sinensis*), grapefruits (*Citrus paradisi*), mandarins (*Citrus reticulata*), clementines (*Citrus clementina*) and other orange‐related species that contain *p*‐synephrine. Mandarin oranges juice may contain more than 20 mg and as much as 40 mg *p*‐synephrine per eight fluid oz glass (Dragull, Breksa, & Cain, 2008; Uckoo, Jayaprakasha, Nelson, & Pati, 2011).

Various *Citrus* cultivars are the plant sources with the highest known concentration of *p*‐octopamine. For example, Meyer lemons and other lemons as well as mandarin oranges are common sources (Uckoo et al., 2011; Wheaton & Stewart, 1969). As a consequence, humans are frequently exposed to *p*‐octopamine. Juices of other *Citrus* species including Marrs sweet oranges, grapefruits, pummelos (*Citrus grandis*), tangerines (*Citrus tangerina*) and clementines contain no detectable *p*‐octopamine. *p*‐Octopamine also occurs in mollusks, other invertebrates and various other animals (Stohs, 2015). In humans, it is produced in the brain and nerve tissues in trace amounts, and may act as a neurotransmitter precursor and neuromodulator as well as a biomarker for neurological disorders (Shi et al., 2016; Stohs, 2015).

Ephedra (*Ephedra sinica*, ma huang) has been used medicinally in China for over 5,000 years (Abourashed, El‐Alfy, Khan, & Walker, 2003; Trease & Evans, 1966). Ephedrine, the primary active constituent in ephedra, is derived from the aboveground parts of the plant and related species, but can also be chemically synthesized. Most prominent sources of ephedra and ephedrine are from China, India and Pakistan (Abourashed et al., 2003). The use of ephedra in dietary supplements is banned in the United States by the FDA, although it can be used in traditional Chinese medicine. As a drug, ephedrine is used to treat or prevent hypotension (Dusitkasem et al., 2017), and has been used for asthma, obesity and narcolepsy (Abourashed et al., 2003; Trease & Evans, 1966).

As noted above, *m*‐synephrine is derived by chemical synthesis and does not occur naturally in *Citrus* or other plant genera (Arbo et al., 2008; Avula, Upparapalli, Navarrete, & Khan, 2005; Mattoli et al., 2005; Mercolini et al., 2010; Nelson, Putzbach, Sharpless, & Sander, 2007; Pellati & Benvenuti, 2007; Pellati, Benvenuti, & Melegari, 2004, 2005; Pellati, Benvenuti, Melegari, & Firenzuoli, 2002; Roman, Betz, & Hildreth, 2007; Tsujita & Takaku, 2007), which is contrary to suppositions in various articles and reviews (Bent et al., 2004; Haaz et al., 2010; Penzak et al., 2001; Rossato et al., 2010; Smedema & Muller, 2008; Stephensen & Sarlay Jr., 2009).

## 3 MECHANISTIC STUDIES

Cardiovascular effects of ligands are associated with direct adrenergic receptor binding and/or through indirect effects as the release of norepinephrine and epinephrine. In general, vasoconstriction occurs when ligands bind to α‐adrenergic receptors, while binding to β‐1 adrenergic receptors result in myocardial contractility and increased heart rate. Ligand binding to β‐2 adrenergic receptors is associated with bronchodilation (Inchiosa Jr., 2011). β‐3 adrenoreceptors are located in white and brown adipose tissues and muscles as well as other tissues, and their activation results in various metabolic effects such as increases in lipolysis, and improvements in insulin resistance, glycemic control and lipid profiles (Coman et al., 2009).

Ephedrine exhibits multiple mechanisms of action consisting of an indirect effect which involves the release of norepinephrine and epinephrine as well as a direct effect on adrenergic receptors (Andraws, Chawla, & Brown, 2005; Diepvens, Westerterp, & Westerterp‐Plantenga, 2007; Haller & Benowitz, 2000; Inchiosa Jr., 2011; Mund & Frishman, 2013). Through the indirect effect of ephedrine, norepinephrine and epinephrine act on α‐1, β‐1, and β‐2 adrenergic receptors to produce cardiovascular affects, while interacting with β‐3 adrenergic receptors to promote thermogenesis (Mund & Frishman, 2013). Ephedrine also acts directly on all these adrenergic receptors to produce thermogenesis and cardiovascular effects (Andraws et al., 2005; Diepvens et al., 2007; Haller & Benowitz, 2000; Inchiosa Jr., 2011).

The ability of ephedrine to act as an adrenergic agonist was studied in rat lung cell membranes (Jiang, Liu, Wang, Zhan, & Shu, 1987). Ephedrine binding was approximately 20‐fold greater than its enantiomeric form pseudoephedrine to adrenergic receptors. No distinction was made between β‐1 and β‐2 receptors (Jiang et al., 1987). The abilities of ephedrine and pseudoephedrine to bind to β‐2 adrenergic receptors have also been studied (Li et al., 2014). The authors concluded that the differences in the association constants accounted for the differences in the pharmacological potencies of the two compounds.

The immunochemical identification of β‐3 adrenergic receptors in various tissues of obese human subjects treated with ephedrine was determined by De Matteis et al. (2002). Ephedrine administration increased the expression of β‐3 adrenergic receptors in obese subjects, with the detection of these receptors in adipocytes and ventricular myocardium as well as smooth muscle of the gall bladder, colon, ileum and prostate. The authors also concluded that the “expression in ventricular myocardium is consistent with the evidence that the β‐3 adrenergic receptor mediates a negative inotropic effect on this tissue”. These results are consistent with the well‐known ability of the ability of ephedrine to suppress appetite and facilitate weight management and weight loss (Hackman et al., 2006).

The role of thioredoxin‐1 expression in the effects of ephedrine was studied in rat pheochromocytoma PC‐12 cells in culture, providing insight into the cellular and molecular mechanisms of action. Thioredoxin‐1 is a redox regulating protein with various biological activities including the regulation of DNA binding transcription factor and consequent neuroprotection. This study also demonstrated that ephedrine induced thioredoxin‐1 expression through a β‐2 adrenergic receptor/cyclic AMP/protein kinase/dopamine‐ and cyclic AMP‐regulated phosphoprotein signaling pathway, but did not involve β‐1 adrenergic receptor binding (Jia, Zeng, Li, Ma, & Bai, 2013).

Brown et al. (1988) observed that [R‐(−)] stereoisomers (l‐forms) of both *p*‐synephrine and *p*‐octopamine were approximately 1,000‐fold less active in binding to rat aorta α‐1 adrenergic receptors and α‐2 adrenergic receptors from rabbit saphenous vein than norepinephrine. *m*‐Synephrine (phenylephrine) binding was 150‐fold and sixfold less, respectively, to these two receptors than norepinephrine. The [S‐(+)] stereoisomers (*d*‐forms) of *p‐*octopamine and *p*‐synephrine exhibited over 100‐fold lower binding actives than the [R‐(−)] stereoisomers (*l*‐forms) to α‐1 and α‐2 adrenergic receptors.

Ma, Bavadekar, Schaneberg, Khan, and Feller (2010) concluded that *p*‐synephrine acts as an antagonist rather than an agonist with respect to human α‐2a‐ and α‐2c adrenergic receptors. Furthermore, *p*‐synephrine was approximately 50‐fold less potent in activating human α‐1a adrenergic receptors. Several studies have concluded that the hydroxyl group in the para position of the ring as occurs in *p*‐synephrine decreases adrenergic receptor binding and the subsequent cardiovascular effects (Ma et al., 2010; Mukherjee, Caron, Mullikin, & Lefkowitz, 1976). Jordan, Thonoor, and Williams (1987) concluded that *p*‐synephrine bound to the β‐1 and β‐2 adrenergic receptor about 10,000‐fold or less actively than norepinephrine in guinea pig atria and trachea.

Carpene' et al. (1999) examined the lipolytic activity of a number of potential β‐3 adrenergic receptor agonists including *p‐*synephrine, *p*‐octopamine and noradrenaline (norepinephrine) in white fat cells from hamsters, rats, dogs, humans and guinea pigs. *p*‐Octopamine was the most selective for β‐3 adrenergic receptors, stimulating lipolysis in rat, hamster and dog adipocytes. *p*‐Octopamine was the only amine the authors studied that fully stimulated lipolysis in rat, hamster and dog fat cells, but was ineffective in human and guinea pig fat cells. *p*‐Synephrine was partially active in stimulating lipolysis in all species while tyramine, dopamine, and *β*‐phenylethylamine exhibited no activity. The authors concluded that *p*‐octopamine was the most selective agonist for β‐3 adrenergic receptors. These studies demonstrated marked differences in adrenergic receptor binding among the various biogenic amines that were assessed.

In a subsequent study, the lipolytic activity of *p*‐synephrine, *p‐*octopamine, tyramine and *N*‐methyltyramine were compared in rat and human adipocytes based on β‐3 adrenergic receptor binding (Mercader, Wanecq, Chen, & Carpene, 2011). In rat fat cells, at a concentration of 10 μg/ml both *p‐*synephrine and *p*‐octopamine exhibited approximately 60% of the lipolytic activity of 1 nM/ml of isoprenaline while tyramine and *N*‐methyltyramine exhibited no effect or were weakly antagonistic. In human adipocytes, 10 μg/ml of both *p‐*synephrine and *p*‐octopamine exhibited approximately 10% of the lipolytic activity of 1 μM/ml of isoprenaline. Various studies indicate that *N*‐methyltyramine acts as an α‐adrenergic receptor antagonist while promoting appetite and inhibiting lipolysis, effects counter to those of ephedrine, *p*‐synephrine and *p‐*octopamine (Stohs & Hartman, 2015).

An extension of previous studies affirmed that the adrenergic receptor binding of *p*‐synephrine and *p*‐octopamine in rodents was at least 10‐fold greater than in humans while tyramine and *N*‐methyltyramine exhibited no binding activity (Carpene', Testar, & Carpene', 2014). In fact, half‐maximal lipolysis stimulation was achieved with a 100‐fold lower dose of *p*‐octopamine in mouse adipocytes as compared to human adipocytes. These results indicate that mice may be much more responsive to *p*‐octopamine than *p*‐synephrine and support previous observations that effects produced in rodents at specific doses cannot be directly extrapolated to humans (Mercader et al., 2011). In this study, high concentrations *p*‐synephrine and *p*‐octopamine were shown to activate glucose transport in human fat cells.

Several studies have examined the effects of *p*‐synephrine on carbohydrate metabolism in perfused rat liver (de Oliveira, Comar, de Sa‐Nakanishi, Peralta, & Bracht, 2014; Peixoto et al., 2012). *p*‐Synephrine increased glycogenolysis, glycolysis, oxygen uptake, glucose output and perfusion pressure. These effects were shown to be at least in part mediated by α‐ and β‐adrenergic signaling, while requiring the simultaneous participation of both cAMP and Ca^2+^ (de Oliveira et al., 2014). The authors concluded that most of the actions of *p*‐synephrine were catabolic.

Neuromedin U2 receptor (NMUR2) is present in the hypothalamic regions of the brain and is involved in the regulation of energy balance, food intake, nociception and stress (Zheng, Guo, Wang, & Deng, 2014). As was demonstrated in NMUR2 negative and short hairpin RNA knockdown HEK293 cell lines, *p*‐synephrine binds to this receptor with high efficacy and potency. The ability of *p*‐synephrine to suppress appetite and enhance eating control has been affirmed in humans (Kaats, Leckie, Mrvichin, & Stohs, 2017) and animals (Arbo et al., 2009). How well *p*‐synephrine can cross the blood brain barrier to achieve functional concentrations and bind to NMUR2 has not been specifically determined, nor have studies been reported regarding the ability of ephedrine, *m*‐synephrine and *p*‐octopamine to across the blood brain barrier. However, ephedrine can be detected in rat brain following its administration (Song et al., 2014) and the neurological effects of ephedrine are well known, thus demonstrating that it is able to cross the blood brain barrier.

In an in vitro study, the effect of *p*‐synephrine on glucose consumption and its mechanism of action were determined in L6 skeletal muscle cells in culture (Hong et al., 2012). *p*‐Synephrine dose‐dependently increased basal glucose consumption by over 50% relative to controls, and had no effect on cell viability. The increased glucose consumption by *p*‐synephrine involved Glut4‐dependent glucose uptake that in turn was dependent upon *p*‐synephrine stimulation of AMP‐activated protein kinase phosphorylation.

The effects of *p*‐synephrine on lipid accumulation and glucose production have been assessed in H411E rat liver cells (Cui, Lee, Lee, & Park, 2014). *p*‐Synephrine dose‐dependently decreased glucose production, and α‐ and β‐adrenergic receptor antagonists did not alter this effect. These results indicated that the effects of *p*‐synephrine on gluconeogenesis did not require involvement of adrenergic receptors.

Several studies have demonstrated the anti‐inflammatory activity of *p*‐synephrine*. p*‐Synephrine suppressed lipopolysaccharide‐induced acute lung injury in mice by reducing the number of inflammatory cells in the lungs, decreasing the levels of reactive species, enhancing superoxide dismutase activity, decreasing tumor necrosis alpha and interleukin‐6 (IL‐6), and increasing IL‐10 (Wu et al., 2014). In normal human fibroblasts and NIH/3 T3 mouse fibroblasts in culture, *p*‐synephrine inhibited IL‐4‐induced eotaxin‐1 expression through the inhibition of signal transducer and activator of transcription (STAT6) phosphorylation which acts as a signal transducer immediately downstream from IL‐4 (Roh et al., 2014). Eotaxin‐1 is a potent chemoattractant and mediator for eosinophils which are associated with inflammation. STAT6 is critical in activating cytokine gene expression and cytokine signaling in immune and target tissue cells. *p*‐Synephrine also inhibited eosinophil recruitment induced by eotaxin‐1 overexpression. *m*‐Synephrine had little effect on eotaxin‐1 induction and therefore little anti‐inflammatory activity. These results indicated that *p*‐synephrine exerts anti‐inflammatory effects at least in part by inhibiting eotaxin‐1 expression (Roh et al., 2014). Arbo et al. (2009) reported that in mouse livers *p*‐synephrine exhibited antioxidant and tissue protective activities by enhancing reduced glutathione content, decreasing glutathione peroxidase activity and increasing catalase activity.

In a study involving isolated adipocytes from rats, Yen, Li, Hsu, Lee, and Cheng (1998) showed that concentrations of 0.01–0.10 nmol *p*‐octopamine activated β‐3 adrenergic receptors to lower glucose uptake into adipocytes and increase cAMP. The involvement of β‐3 adrenergic receptors was confirmed by using a β‐3 adrenergic receptor specific antibody, a specific agonist of β‐3 adrenergic receptors (BRL37344), and the β‐adrenergic antagonists pindolol and propranolol.

In a study in isolated rat fat cells, the lipolytic activity of *p*‐octopamine and tyramine were shown to be approximately 100‐fold less than norepinephrine (Nakano, Ishii, Cole, & Oliver, 1969). *p*‐ Octopamine failed to exhibit β‐adrenergic activity in rats as determined by initiation of thirst and increase in tail skin temperature (Fregly, Kelleher, & Williams, 1979). The relative α‐adrenergic activity of *p*‐octopamine was 2,000‐fold less than norepinephrine (Fregly et al., 1979). In a study involving contractile response of rat vascular smooth muscle, the potencies of *p‐*octopamine and *m‐*synephrine relative to norepinephrine were determined to be 400‐fold less active and about one‐third as active, respectively, clearly demonstrating the greater adrenergic activity of *m‐*synephrine than *p*‐octopamine (Ress, Rahmani, Fregly, Field, & Williams, 1980).

A group of G protein‐coupled receptors known as trace amine‐associated receptors (TAAR) have been identified in recent years in various human and animal tissues, and serve as neuromodulators (Berry, Gainetdinov, Hoener, & Shahid, 2017; Borowsky et al., 2001; Bunzow et al., 2001; Gainetdinov, Hoener, & Berry, 2018; Khan & Nawaz, 2016; Pei, Asif‐Malik, & Canales, 2016; Rutigliano, Accorroni, & Zucchi, 2018). Because they are present in much smaller amounts than the predominant neurotransmitters, the amines which interact with these receptors are referred to as “trace amines”. The most prominent biogenic amines which interact with TAAR include *p*‐octopamine, tyramine, tryptamine and β‐phenylethylamine (Pei et al., 2016; Rutigliano et al., 2018), although *N*‐methyltyramine, *p*‐synephrine and 3‐iodothyronamine have also been included as trace amines (Khan & Nawaz, 2016). Ephedrine is not considered a trace amine and is not found in the human nervous system. Whether it interacts with TAARs is not known.

Humans possess six functional isoforms (subtypes) of TAAR, namely, TAAR1, TAAR2, TAAR5, TAAR6, TAAR8 and TAAR9 (Gainetdinov et al., 2018). Of these isoforms, TAAR1 has been the most extensively studied and may be the most important (Pei et al., 2016; Rutigliano et al., 2018). TAAR1 has been shown to be a neuromodulator of dopaminergic, serotonergic and glutamatergic neurotransmission, and thus has profound physiological, pathophysiological and pharmacological implications (Gainetdinov et al., 2018; Pei et al., 2016; Rutigliano et al., 2018).

The TAARs constitute another mechanism whereby *p*‐synephrine and *p*‐octopamine as well as *N‐*methyltyramine and tyramine may exert various physiological and pharmacological effects either by acting as neurotransmitter precursors or neuromodulators, and serve as biomarkers. For example, the circulating levels of *p*‐synephrine are increased in Parkinson's disease patients while norepinephrine levels are decreased as compared to normal healthy individuals (D'Andrea et al., 2019).

The potential mechanism of action of trace amines and the possible role of TAARs has been studied in porcine coronary and mesenteric arteries (Koh, Chess‐Williams, & Lohning, 2019). The authors concluded that contractile responses in coronary artery involved activity of α1‐adrenergic receptors and TAARs other than TAAR‐1. In contrast, the contractile responses of trace amino acids on the mesenteric artery appeared to involve indirect sympathomimetic activities and direct action on α1‐adrenergic receptors. Concentrations of 10^−3^ and 10^−4^ M of *p‐*synephrine, *p*‐octopamine and tyramine used in this study were up to 15–20 fold greater than blood levels of approximately 10 ng/ml as determined by high performance liquid chromatography following a typical 50 mg dose of *p*‐synephrine (Shara, Stohs, & Mukattash, 2016), and therefore, non‐physiological. No cardiovascular or other adverse effects have been observed in over 30 controlled human studies involving typical oral doses of *p*‐synephrine in the range of 25–100 mg (Stohs, 2017).

The above described in vitro studies indicate that *p*‐synephrine and *p*‐octopamine exhibit effects involving a variety of mechanisms in addition to selective binding to some adrenergic receptors with limited involvement of α‐ and β‐1 and β‐2 adrenergic receptors.

## 4 DISCUSSION AND CONCLUSIONS

The above studies indicate that *p*‐synephrine and *p*‐octopamine cannot be equated with *m*‐synephrine or ephedrine and the effects of ephedrine cannot be extrapolated to *p*‐synephrine or *p*‐octopamine due to structural and stereochemical differences which greatly alter receptor binding characteristics, pharmacokinetic properties and the pharmacological/physiological effects produced. At the doses commonly used, *p*‐synephrine and *p*‐octopamine do not produce adverse effects such as an increase in heart rate or blood pressure that are characteristic of ephedrine and possibly *m‐*synephrine (Ratamess et al., 2018; Shara, Stohs, & Smadi, 2017; Stohs, 2017; Suntar et al., 2018).

The structural differences result in marked differences in pharmacokinetic properties. For example, first pass extraction of *p*‐synephrine is greater than *p*‐octopamine following oral administration (Da Silva‐Pereira et al., 2016). The half‐life of ephedrine following oral administration in humans is approximately 6–7 hr (Csajka, Haller, Benowitz, & Verotta, 2005; Pickup, May, SSendagire, & Paterson, 1976), while the half‐lives of *p*‐synephrine (Hengtmann & Aulepp, 1978; Haller et al., 2005, 2008) and *m*‐synephrine (Golette, 2018; Golette & Zimmerman, 2015) are 2–3 hr and 1–2 hr, respectively. No studies on *p*‐octopamine half‐life were found.

Sympathomimetic agents vary broadly in their abilities to activate adrenergic receptors, and therefore it should not be assumed that substances with some structural similarity will have similar effects (Westfall & Westfall, 2014). Human and animal studies have shown that adverse effects on blood pressure and heart rate are not associated with *p*‐synephrine at commonly used doses (Ratamess et al., 2018; Shara et al., 2017; Stohs, 2017; Suntar et al., 2018) The lack of cardiovascular effects in association with *p*‐synephrine and *p*‐octopamine are due to the fact that both *p*‐synephrine and *p*‐octopamine bind much more poorly to α‐1, α‐2, β‐1 and β‐2 adrenergic receptors than other adrenergic agonists as ephedrine, norepinephrine and *m‐*synephrine, and also exhibits poor indirect effects (Stohs, 2017; Stohs & Badmaev, 2016; Stohs, Preuss, & Shara, 2011). In addition, as described above, there are other differences in the mechanisms of action of *p‐*synephrine, ephedrine and *m*‐synephrine. The effects of *p‐*octopamine are similar to *p*‐synephrine (Marles, 2011).

Various studies have shown that *p*‐synephrine binds to β‐3 adrenergic receptors, resulting in an increase in the body's ability to breakdown fats (Carpene' et al., 1999; Carpene' et al., 2014; Mercader et al., 2011). Binding to β‐3 adrenergic receptors does not influence heart rate or blood pressure, although it may be speculated that cardiovascular down‐regulation due to β‐3 adrenergic receptor binding may result in small decreases in diastolic blood pressure, which has been demonstrated (Ratamess et al., 2018; Shara et al., 2016). Because *p*‐synephrine exhibits little or no binding to α‐1, α‐2, β‐1 and β‐2 adrenergic receptors, cardiovascular effects as an increase in heart rate and blood pressure are not experienced at commonly used doses of *p*‐synephrine, unlike a number of other phenylethylamine and phenylpropylamine derivatives including ephedrine.

Because *p*‐synephrine and *p*‐octopamine bind at least 10 times more readily to α‐1, α‐2, β‐1 and β‐2 adrenergic receptors from rodents than humans (Carpene' et al., 1999; Carpene’ et al., 2014; Mercader et al., 2011), the small but clinically insignificant cardiovascular effects seen in rodents at high doses (Hansen et al., 2012, 2013; Hansen, Juliar, White, & Pellicore, 2011) cannot be extrapolated to humans. As a consequence, based on these receptor binding studies, cardiovascular effects are not predicted or expected to occur in humans.

It is of interest that the U.S. FDA has placed *p‐*octopamine, *N*‐methyltyramine and hordenine on its list of ingredients that do not appear “to be lawful ingredients in dietary supplements” (FDA, 2019). The reason for this designation is not clear, particularly in light of the fact that safety does not appear to be an issue. Bitter orange extracts standardized to *p*‐synephrine may contain small amounts of the minor protoalkaloids *p*‐octopamine, *N*‐methyltyramine, tyramine and hordenine typically in amounts of approximately 0–1, 2–3, 0–1 and 0–1%, respectively, of the total protoalkaloidal content (Stohs, 2015). Thus, the sum of these minor alkaloids represents less than 6% of the total protoalkaloidal content of extracts. Furthermore, as previously noted, *p*‐octopamine occurs widely in citrus specie with the most common source being lemons (*Citrus limon*) Uckoo et al., 2011). As a consequence, humans are widely exposed to *p*‐octopamine with no known adverse effects.

The presence of *N‐*methyltyramine and hordenine in germinated barley is well known, and they have been shown to occur in various beers in the ranges of 0.6–4.6 and 1.0–6.3 mg/L, respectively (Sommer et al., 2019). Therefore, these two protoalkaloids are very widely consumed throughout the world with no known adverse effects. Hordenine showed no changes in heart rate, respiratory rate, body temperature or behavior when given orally at a dose of 2 mg/kg to horses (a dose of 1,000 mg for an average 500 kg horse). Therefore, no effect would be projected in a human that consumed several mg of *N*‐methyltyramine and hordenine from a typical dose of an average beer or a standardized bitter orange extract.

*N‐*Methyltyramine is rapidly absorbed and undergoes N‐demethylation to tyramine followed by rapid oxidative deamination. *N*‐Methyltyramine and tyramine have no effect or are both weak adrenergic antagonists (inhibitors) with respect to fat metabolism and as compared to *p‐*synephrine and *p*‐octopamine which exhibit adrenergic agonist activity (Mercader et al., 2011). The indirect sympathomimetic effects of tyramine have been well demonstrated in animal and in vitro studies (Khwanchuea, Mulvany, & Jansakul, 2008; Koh et al., 2019). However, no adverse effects have been observed after dietary exposure to 600 mg tyramine in normal healthy individuals (EFSA, 2011). Tyramine has an LD_50_ in rats greater than 2000 mg/kg (Til, Falk, Prinsen, & Willems, 1997), indicating a low acute toxicity.

How exogenously administered *p*‐octopamine and *p*‐synephrine influence TAARs is not clear. Because both undergo very rapid and extensive hepatic first pass extraction and metabolism (da Silva‐Pereira et al., 2016), it is very possible that small amounts of orally ingested *p‐*synephrine and *p‐*octopamine reach neurological tissues and TAARs. The rapid extraction and metabolism may also account for the lack of observed adverse effects.

In summary, small structural and stereochemical differences between *p*‐synephrine, *p*‐octopamine, *m*‐synephrine and ephedrine as well as epinephrine and norepinephrine result in markedly different receptor binding and physiological/pharmacological properties. Therefore, the effects associated with one of these compounds cannot be extrapolated to others.

## CONFLICT OF INTEREST

SJS has served as a consultant for Innophos LLC. SDR and MS have no potential conflicts of interest to report.
